# A Directory of Dentists

**Published:** 1880-06

**Authors:** 


### EDITORIAL.

### A DIRECTORY OF DENTISTS.

At the last annual meeting of the American Dental Association, the following resolutions were presented and adopted:

*Resolved* That a committee, consisting of one member of the Association from each State, and from the District of Columbia, be appointed by the President to make (with the assistance of the State societies) a complete directory of the dentists of the United States.

*Resolved.* That the members of the committee be directed to bring the project before their several State and district societies, in order that such action may be taken as will rectify the directory each year.

*Resolved.* That when any State or district society shall present to this Asscciation a list, alphabetically arranged, of dentists within such State or district, then this Association shall, through its Committee on Directory, present to such State or district society all lists of dentists in its possession, and shall continue to exchange the amended lists as long as such shall be continued by the several State and district societies.

For carrying out the objects of these resolutions, the following committee was appointed; D. C. McNaughton, Jersey City, N. J., *Chairman*:

Thos. Fillebrown..........Maine.
W. E. Page..........New Hampshire, Vermont and Massachusetts.
C. A. Brackett..........Rhode Island.
J. McManus..........Connecticut.
A. A. Freeman..........New York.
J. E. Registre..........Delaware.
G. W. Klump..........Pennsylvania.
F. J. S. Gorgas..........Maryland.
R. Finley Hunt.......... District of Columbia.
J. H. Moon..........Virginia.
D. B. McLane..........W. Virginia.
E. Floyd..........N. Carolina.
Thos. T. Moore..........S. Carolina.
R. F. Phillips..........Florida.

| | |
|---|---|
| G. W. M. Whitaker | Georgia. |
| E. S. Chisholm | Alabama. |
| J. D. Mills | Mississippi. |
| G. J. Frederics | Louisana. |
| J. A. Lulloss | Texas. |
| E. Collins | Arkansas. |
| A. H. Thompson | Kansas. |
| H. W. Morgan | Tennessee. |
| A. O. Rawls | Kentucky. |
| A. F. Enninger | Ohio. |
| S. B. Brown | Indiana. |
| Geo. L. Field | Michigan. |
| Arthur Holbrook | Wisconsin. |
| D. C. Price | Minnesota. |
| L. C. Ingersoll | Iowa. |
| T. C. Kern | Nebraska. |
| J. C. Fuller | Colorado. |
| W. C. Conwell | Nevada. |
| Wm. Dutch | Calafornia |
| S. J. Barber | Oregon. |
| J. G. Hanfre | Missouri. |
| E. F. Darby | Territories at Large. |

This is a desirable object and one to which State societies can profitably give some attention. In what way, it might be asked, will such a directory benefit the profession? And first it may be answered, It will be gratifying to everyone interested in the profession to know its numerical proportions and the distribution in the different parts of the country; it will be interesting to study the influences modifying such distribution.

Such a directory is especially interesting to the dental society of each State; it confers an influence and a favor that cannot be in any other way attained; it enables such societies to more fully utilize and bring into active co-operation the better part of the profession within the respective States.

Such a work in States that now have societies will stimulate others to organize associations and so to enter upon the general work of association effort.

Such a directory will be valuable to all who, for any purpose, desire to reach a large number of the profession.

It will be a means of facilitating communication between the members of the profession generally, and thus bring them into closer and more harmonious relations, and afford a better opportunity for co-operation.

Three of the States, viz., Ohio, Michigan and Kentucky, already have very complete and accurate lists of the dentists in each. Ohio obtained a list in 1876 and had it revised in 1879. Michigan and Kentucky in 1878; these were all very full and accurate at the time they were obtained; frequent revision is necessary to retain accuracy. The details of this work cannot be taken up by the American Dental Association, but must be left in the hands of the State organizations. Any stimulation or fostering care by the former may be well, but for this body to attempt to secure and keep an accurate and full list of the profession of the United States would require the employment of a good book-keeper and register through the year, and would add greatly to the present financial burdens of the society. There are certain interests that would be greatly benefitted by such a directory, viz colleges, publishers, dealers, inventors and manufacturers. By co operation these parties might ere this have had a very perfect work of this kind. Individuals have done something in this direction on their own account, but there has been no general effort.

It will be observed that some of the persons on the above named committee are not members of the American Society, and in some instances not of any State society. But the selection of such persons was made with the hope that they would be interested and co-operate through their respective State societies; and even if they have no such society, that they might become stimulated to secure such an organization. Every State in the Union ought to have its dental society, and should be represented in the central organization.

It is suggested to those members of the committee residing in States having no society, that they make on effort to organize; and if that cannot be done, then call together a sufficient number of the profession who can co-operate in carrying out the objects of these resolutions.

It would be gratifying indeed if one-half, or more, of the State societies would come up to the next meeting of the Association with their respective Directories completed. *So may it be.*

